# Intraoperative Full-Dose of Partial Breast Irradiation with Electrons Delivered by Standard Linear Accelerators for Early Breast Cancer

**DOI:** 10.1155/2014/568136

**Published:** 2014-12-17

**Authors:** Alfredo Carlos S. D. Barros, Samir A. Hanna, Heloísa A. Carvalho, Eduardo Martella, Felipe Eduardo M. Andrade, José Roberto M. Piato, José Luiz B. Bevilacqua

**Affiliations:** ^1^Mastology Center, Hospital Sírio Libanês, Rua Dona Adma Jafet 91, 01308-050 São Paulo, SP, Brazil; ^2^LIM 02, Discipline of Human Structural Topography, University of São Paulo Medical School, Avenida Dr. Arnaldo 455, 01246-903 São Paulo, SP, Brazil; ^3^Department of Radiotherapy, Hospital Sírio Libanês, Rua Dona Adma Jafet 91, 01308-050 São Paulo, SP, Brazil; ^4^Radiotherapy Service, Hospital das Clínicas, University of São Paulo Medical School, Rua Dr. Enéas de Carvalho Aguiar 225, 05403-000 São Paulo, SP, Brazil; ^5^Hospital Perola Byington, Avenida Brigadeiro Luís Antônio 683, 01317-000 São Paulo, SP, Brazil

## Abstract

*Purpose*. To assess feasibility, efficacy, toxicity, and cosmetic results of intraoperative radiotherapy (IORT) with electrons delivered by standard linear accelerators (Linacs) during breast conserving surgeries for early infiltrating breast cancer (BC) treatment. *Materials and Methods*. A total of 152 patients with invasive ductal carcinoma (*T* ≤ 3.0 cm) at low risk for local relapses were treated. All had unicentric lesions by imaging methods and negative sentinel node. After a wide local excision, 21 Gy were delivered on the parenchyma target volume with electron beams. Local recurrences (LR), survival, toxicity, and cosmetic outcomes were analyzed. *Results*. The median age was 58.3 years (range 40–85); median follow-up was 50.7 months (range 12–101.5). There were 5 cases with LR, 2 cases with distant metastases, and 2 cases with deaths related to BC. The cumulative incidence rates of LR, distant metastases, and BC death were 3.2%, 1.5%, and 1.5%, respectively. Complications were rare, and the cosmetic results were excellent or good in most of the patients. *Conclusions*. IORT with electrons delivered by standard Linacs is feasible, efficient, and well tolerated and seems to be beneficial for selected patients with early infiltrating BC.

## 1. Introduction

It is well known that whole breast irradiation (WBI) after breast conserving surgeries for patients with early infiltrating breast carcinoma (BC) significantly reduces the likelihood of local recurrence (LR) [1]. There are several evidences that LR is a predisposing factor for systemic metastasis [2–4] and, within this scope, radiotherapy (RT) is very useful for treating residual tumor cells after the surgery. The most used schedule for WBI is 50 Gy delivered in 5 weeks using conventional fractionation.

There is no consensus, however, regarding whether the entire breast needs to be irradiated [5]. The accelerated partial breast irradiation (APBI) concept, based on confining the irradiation to the vicinity of the tumour bed, shortening the course of the treatment and allowing more convenience for patients, has contributed to changes in the RT paradigms [6, 7].

A variety of APBI techniques, including low- or high-dose rate brachytherapy, balloon brachytherapy, localized external beam RT (using either three-dimensional or intensity-modulated), and intraoperative electron or photon beam treatments, have been used with encouraging results [8–13].

Nondedicated linear accelerators (Linacs) capable of delivering treatment with electrons have been used for intraoperative irradiation of many other tumors [14, 15]. These types of equipment are available in almost every RT facility and are used for daily patients' treatments. The possibility of delivering intraoperative radiotherapy (IORT) with electrons, without dedicated equipment, is very attractive. Addressing this issue, the purpose of our paper was to assess the efficacy, toxicity, and cosmetic outcomes of IORT delivered by standard Linacs, during breast conserving surgeries for the treatment of early breast cancer.

## 2. Materials and Methods

A prospective phase II cohort study started in May 2004 at the Sirio Libanes Hospital in Sao Paulo, Brazil. As of July 2012, 187 women with diagnosis of BC by percutaneous biopsy were enrolled. The research protocol was approved by the Ethics Committee of the hospital.

Patients were eligible if they had unicentric invasive ductal carcinoma, with less than 3.0 cm at the largest diameter confirmed by mammography, ultrasonography, and magnetic resonance imaging (MRI).

Patients were considered ineligible if any of the following features were present: skin involvement, history of BC in the contralateral breast, or intraoperative microscopic findings of involvement of surgical margins or sentinel node (SN). Invasive lobular carcinoma subtype was also an exclusion criterion due to its high rate of multicentricity and multifocality.

### 2.1. Surgery

The breast conserving surgeries were performed at an operating theater located inside the Radiotherapy Department, contiguous to the Linac suite. The quadrantectomy consisted of an “en bloc” resection of the parenchyma and pectoralis fascia, with at least a 2 cm macroscopic margin around the tumor. The skin over the tumor was generally removed by a circular incision, with its conservation being possible in small, deeply located tumours (*T* ≤ 1.0 cm).

After verification of clear margins by intraoperative histopathologic and cytologic exams, SN radioguided biopsy was generally performed by the unique breast incision, as previously described [16, 17]. SN was analyzed by means of cytology.

As for surgical aspects, the same maneuvers standardized for electron intraoperative therapy (ELIOT) by Veronesi et al. were adopted [9, 18]. Once the wide local excision and the SN biopsy were performed, the glandular tissue was detached from the pectoralis major muscle, to an extension of 3 cm margin around the resected area, and the skin flaps were detached from the parenchyma at the level of the adipose lamina for 2 cm circumferentially.

The surgical bed was filled with a wet compress, the wound was covered, and the patient was transferred to the RT room, where all of the materials needed for maintaining anaesthesia, including gases, were available.

A three-layer disk made of lead (down), aluminium (middle), and silicon (up) was inserted underneath the gland over the muscle, to protect the normal tissue below the irradiated area and absorb the backscattered radiation. The shielding disks (0.5 cm thick each) were available in three diameters (6, 8, or 10 cm), and the largest one fitting the space was placed. The parenchyma was approximated over the disk by separated stitches, exposing the area to the electron beam.

### 2.2. Radiotherapy

Irradiation was performed using one of two standard models of Siemens linear accelerators: Primus or KD2. Both machines produce electrons and are able to generate photon and electron beams with energy ranging between 6 and 21 MeV. A single total dose of 21 Gy prescribed at the 90% isodosis was delivered directly to the parenchyma at a rate of 300 cGy/min.

**Figure 1:**
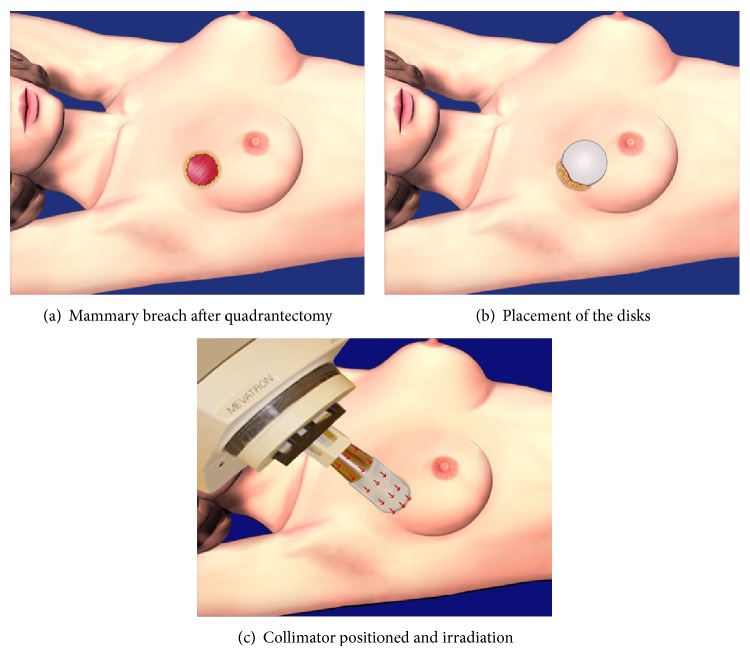
Preparation for delivering irradiation.

The electron beam energy was chosen after measuring the gland thickness by inserting a needle perpendicularly to the parenchyma. A sterile, round collimator was connected to the gantry of the Linac and gently placed into the surgical bed by appropriated mobilization of the couch and gantry (Figure 1). The choice of collimator diameter was made according to each case but was usually up to 6 cm.

**Figure 2:**
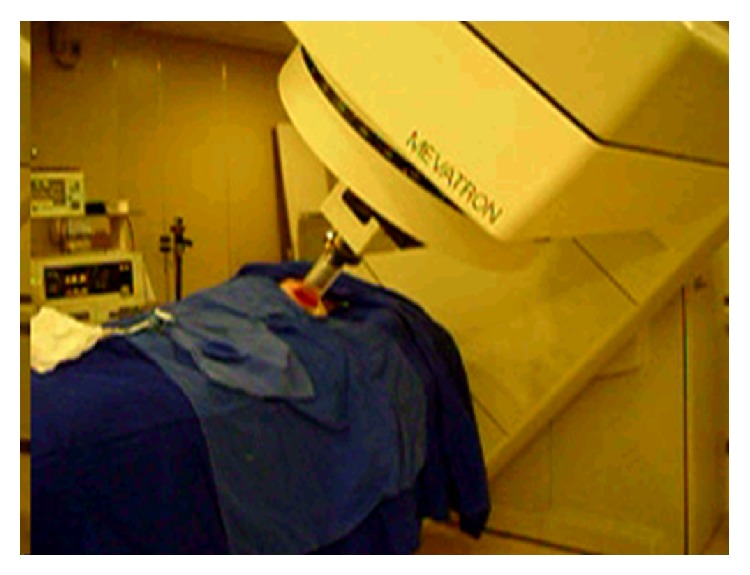
Intraoperative irradiation.

A portal film was taken placing the film below the accelerator couch, orthogonally to the collimator, to guarantee the exact positioning of the disks. This procedure was repeated, if necessary, until the disk was considered well positioned. Afterwards, the staff left the room; the irradiation was delivered during in average 8 minutes, according to the chosen energy, under video surveillance of the vital signs of the anaesthetized patient (Figure 2). Subsequently, the collimator and the disk were removed. The breast tissue was then reconstructed using oncoplastic techniques, preferentially outside the Linac room, in the operating room [19–21].

The whole irradiation procedure lasted approximately 30 minutes, including patient transfer.

### 2.3. Adjuvant Treatment and Follow-Up

Adjuvant systemic therapy was at the discretion of the physician, in accordance with current guidelines [22]. More than half of the patients received hormone therapy alone (51.3%), 8.5% of the patients received only chemotherapy, 38.1% had both, and 1.9% patients had no adjuvant systemic therapy.

Follow-up was performed every 3 months in the first year and every 6 months thereafter. Mammography and ultrasonography were performed at the 6-month visit and annually thereafter.

The primary outcome of the study was LR as the first unfavorable event. Secondary outcomes were local toxicity and cosmesis.

LR was considered as a true relapse (TR), which represents regrowth of residual malignant cells in the same region of the primary tumour, or a second primary tumour (SPT), representing tumor growth in another quadrant, suggesting a distinct clonal origin.

The presence of seromas, hematomas, fat necrosis, wound infections, and dehiscences was investigated at all time points after surgery. Events that occurred until one month after the treatment were considered as “early” and after 6 months as “late.”

Cosmesis evaluation was scored by the physician at least 12 months after irradiation, in accordance with the Harvard criteria [23]. Briefly, the treated breast is compared with the contralateral one and the result is classified as excellent (minimal or no difference in the size or shape); good (mild asymmetry in the size or shape); fair (obvious differences in the size and/or shape); and poor (marked change in the appearance involving more than 1/4 of the breast).

### 2.4. Statistical Analysis

Descriptive and frequencies analysis were performed. The cumulative incidence of LR, overall survival, and BC survival were calculated using the Kaplan-Meier method. The SPSS package version 17.0 (Chicago II) MedCalc package, 11.3.3.0 version (Mariakerke, Belgium), was used for statistical analysis.

## 3. Results

Of the 187 enrolled patients, 35 (18.7%) were intraoperatively excluded because of SN positivity (18 patients), difficulty in obtaining clear margins (11 patients), multicentricity/multifocality (3 patients), muscle infiltration (1 patient), *T*≻3.0 cm (1 patient), and no SN identification (1 patient).

A total of 152 patients received IORT with electrons and were analyzed. The median age of the patients was 58.3 years (range 40–85).  Table 1 summarizes the patients and tumours characteristics. The median follow-up time was 50.7 months (12–110.5).

**Figure 3:**
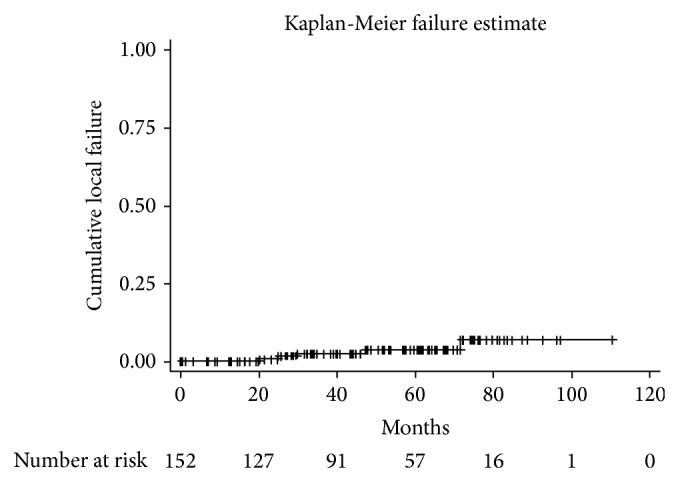
Kaplan-Meier cumulative local failure estimate.

Five of the 152 patients presented LR (TR or SPT). The cumulative incidence of LR as the first unfavorable event was 3.2% (95% CI: 0.8–8.1) (Figure 3). Among the 5 cases of LR in the entire cohort 4 were considered to be a TR, and one had a failure in a quadrant other than the index lesion at 30-month follow-up, consistent with SPT. Regarding other failures, two patients (1.3%) developed distant metastases, three had axillary failure (1.9%), and one patient had a contralateral tumor (0.6%). It is worth noting that among the cases with TRs one had SN micrometastasis and one had lobular carcinoma, both identified only in the definitive histopathological analysis.

One hundred and nine cases were followed up for at least 36 months with an estimated LR rate of 4.6%. Thus, the TR cumulative incidence at 36 months was 2.6%. Kaplan-Meier estimates of efficacy at three years were LR of 4.6%, contralateral breast tumor 0.9%, distant failure 1.8%, cancer specific survival 98.2%, and overall survival 98.0%. The cumulative incidences of first unfavorable events are outlined in Table 2, and the 3-year actuarial rates of recurrences are presented in Table 3.

**Figure 4:**
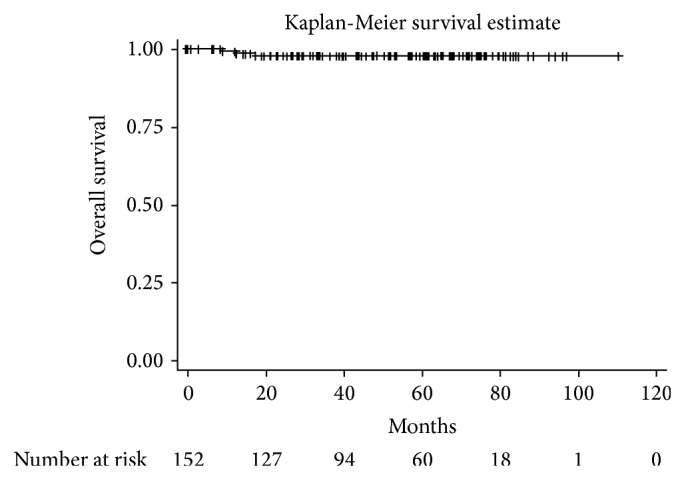
Kaplan-Meier overall survival estimate.

There were three deaths (1.9%): two related to breast cancer (one secondary to pulmonary metastasis and another due to chemotherapy toxicity) and one nononcologically related death. Overall survival is shown in Figure 4.

In the first month after surgery, 6 cases of skin erythema (3.9%), 2 wound dehiscences (1.3%), and 1 case of hematoma (1.9%) were observed. These events were considered as early postoperative complications. Evidence of late toxicity, observed after at least 6 months of follow-up, was seen in 45 patients (29.6%) in a median time of 8 months (range 8–24). There were 21 cases (13.8%) of breast fibrosis (13 mild and 8 severe) and 15 cases of fat necrosis (9.8%). Among these cases, 6 patients required punctions and other 6, surgical drainage. There were also 3 cases of breast lymph edema and 2 cases of nipple retraction.

**Figure 5:**
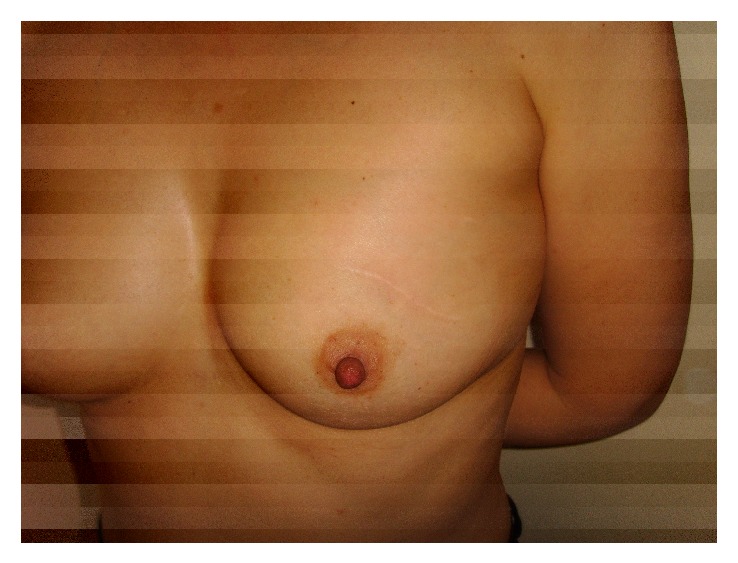
Excellent esthetic result 4 years after the procedure.

The esthetic outcomes have shown 70.3% excellent, 14.4% good, 3.9% regular, and 3.2% of bad results. From the entire cohort, 7.8% of patients were not cosmetically evaluated. Cosmetic outcomes are listed in Table 4. In Figure 5 a case with an excellent esthetic result four years after the procedure is shown.

## 4. Discussion

Breast conserving surgery followed by external WBI is a well established treatment for most women with early infiltrating BC [24, 25]. Currently, more sophisticated RT techniques are available, allowing better target coverage with better normal tissue sparing [26]. In this context, APBI is a rapidly evolving strategy, with a widespread support for its use [7, 27, 28].

The main biologic rationale for intraoperative partial breast irradiation is that 85% of the LR (almost 100% of TR) occurs in the vicinity of the tumour, next to the scar, as a consequence of the persistence of neoplastic cells that most likely possess aggressive cancer stem cell properties [29, 30]. Experimental data indicate hierarchical organisation of BC with a small number of cancer-initiating cells (CICs) that have ability to self-renew and exhibit multilineage potential [31]. CICs, in contrast to their tumorigenic counterparts, can survive fractions of sublethal doses of RT, retaining self-renewal capacity over several generations [32–34]. Some properties of CICs could make them a more vulnerable target to a single lethal irradiation dose, soon after the breast resection, without allowing postoperative hypoxia and time for cell repopulation [35]. Effects of IORT on tumor microenvironment could improve outcomes, as it impairs local proliferation caused by surgical manipulation, inflammation, and simulation of the epithelial mesenchymal transition [36, 37].

Different RT techniques can be used with this purpose and, given that intraoperative RT with standard Linacs has previously been used to treat abdominal tumors, we decided to use this form of treatment during breast conserving surgeries. The surgeries were performed at an operating theatre in the RT department, close to the Linac room where the patients were transferred to receive the irradiation. This geographic characteristic by itself turned out to be a feature that helped the better feasibility of the method. However, it is still possible to transport the patient from an operative theatre far from the Linac suite (usually out of business hours), previously prepared to be used as an operating room [38].

The patient transport from the operating room to the Linac may be regarded as a disadvantage of the method, when compared to the treatment with a dedicated Linac. But one must realize that the use of a nondedicated Linac, mainly in developing countries, may represent a cost-benefit strategy. We have previously reported our outcomes with focus on technical aspects, and the highlights of the use of nondedicated machine were to explore its capability of producing higher electron beam energies rather than dedicated machines and to check the possibility of misalignment between the collimator and the shielding disks by obtaining portal films using photon beams [39].

Other advantages of IORT with electrons are accurate targeting of RT and a precise definition of the tumour bed volume under direct guidance, offering very good dose homogeneity and more effectively sparing of the heart and lungs when compared to external beam RT [35].

At the moment there are two published randomised trials focusing on single dose of RT during breast-conserving surgeries. Vaidya et al., using localized photon beams delivered by the Intrabeam device (Carl Zeiss Meditec, Oberkochen, Germany), concluded that such approach is as efficient as conventional fractioned external beam RT for carefully selected patients [40]. Veronesi et al. did a study with ELIOT at the European Institute of Oncology [41]. They employed two types of dedicated linear accelerators: NOVAC 7 (Hitesys, Latina, Italy) and Liac (Info and Tech, Rome, Italy). Although they found that the rate of LR in the ELIOT group was within the prespecified equivalence margin of results, it was observed that this rate was significantly greater than with external radiotherapy, pointing out the necessity of defining the optimal patient selection criteria.

By far, the most important benefit of IORT with electrons is shortening the RT duration from the traditional 5-6 weeks to 5–8 minutes, thereby eliminating the delay in receiving RT, alleviating emotional distress, avoiding logistical difficulties in travelling to the radiation facility and ensuring 100% compliance. The rate of undertreated patientsdue to incomplete fractioned adjuvant WBI is far from ideal, especially in developing countries, being such women exposed to a higher risk of BC recurrence [42, 43].

The key feature for the development of ELIOT by the Italian group was the estimation of dose equivalence between the standard 60 Gy divided into 30 fractions and the single dose of 21 Gy [44]. In a landmark paper, Veronesi et al. presented a large phase II study that included 1,822 cases treated with ELIOT using dedicated machines [45]. After a mean follow-up period of 36.1 months, 42 women (2.3%) developed a TR, 24 women (1.3%) had a new primary ipsilateral tumour, and 26 women (1.4%) had distant metastases as the first event. Five- and 10-year survival rates were 97.4 and 89.7%, respectively. Compared with conventional RT, ELIOT was considered a safe procedure for women with tumours measuring less than 2.5 cm, with a slightly higher LR rate. Our results presented here are very similar to the results obtained by Veronesi et al.

The widespread use of APBI motivated the American Society of Therapeutic Radiology and Oncology to define a suitable group of patients for whom APBI is acceptable outside of clinical trials, including the following: women older than 60 years, with T1 IDC, clear margins, and the absence of multicentricity, multifocality, and axillary nodes involvement [27]. The European Society for Therapeutic Radiology and Oncology also proposed suitable conditions for APBI: age ≥ 50 years, unicentric and unifocal *T*_1-2_ (≤3.0 cm), pN0 nonlobular invasive cancer, the absence of an extensive intraductal component and lymphovascular invasion, and negative surgical margins of at least 2 mm [28]. Currently it is also known that estrogen receptor negativity is associated with increased risk of LR following APBI [46].

This study has started before the publication of these recommendations, and part of our cases should be considered not suitable for APBI. However, some other results have pointed out that even patients who do not meet the ideal conditions may be locally treated with success [47, 48]. Anyway, since the publication of the recommendations (2009), women under 50 years of age were no longer accepted in our study.

Although it might be tempting to offer IORT to a large number of patients, at this time, a careful selection of suitable patients is paramount. For this reason we advocate preoperative MRI which was performed in all of our patients, to better select the cases for partial breast irradiation. Most likely, the traditional WBI reduces the rate of SPT in the treated breast solely if they were present and occult at the time of the primary treatment. MRI could potentially contribute to the more precise detection of multifocal or multicentric disease, with improvement of operative outcomes and decreased recurrence rates [49], although, besides the MRI high diagnostic accuracy, it is always desirable to have pathological verification of the findings because of the MRI high false-positive rates [50].

The confirmation of intraoperative clear surgical margins is also mandatory, since the objective of IORT is to reduce LR by treating residual malignant cells that may persist in tumour-bearing areas.

With regard to efficacy, the incidence of LR in this cohort was low and acceptable. Moreover, complications due to local toxicity were scarce, and this form of IORT led to a favorable impact on body image, as already observed by other authors [51]. Also, when oncoplastic maneuvers are required, including immediate breast reconstruction with prostheses, they are feasible and safe [19, 21].

We consider as limitations of this study the facts that there was not a control group and that it was performed at a single institution on relatively small number of patients. In spite of these caveats, the technique was demonstrated to be feasible and was successfully implemented, with a very short learning curve. IORT with electrons delivered by conventional Linacs, immediately after a wide local excision, presented the expected results until now, with very good local control and cosmetic outcomes and a low toxicity rate. Selected patients with early infiltrating breast carcinomas may benefit from the technique, which may represent an interesting option for developing countries.

## Figures and Tables

**Table 1:** Characteristics of the patients.

	*n*	(%)
Age (years)		
<50	45	29.6
50–59	39	25.6
≥60	68	44.7
Menopausal status		
Before menopause	36	23.6
After menopause	116	76.4
Tumor size		
pT1	133	87.5
pT2	19	12.5
Estrogen receptor		
Positive	140	92.1
Negative	12	7.9
Progesterone receptor		
Positive	140	92.1
Negative	12	7.9
Grading		
G1	14	9.2
G2	79	51.9
G3	59	38.8

**Table 2:** Incidence of first unfavorable events.

Event	*n*	%
Local recurrence	5	3.3
True local recurrence	4	2.6
Second primary tumor	2	1.3
Axillary relapse	2	1.3
Distant metastasis	2	1.3
Contralateral tumor	1	0.6

**Table 3:** The 3-year actuarial rates of recurrence. Data of the 109 patients with at least 36 months of follow-up.

Pattern of failure	3-year actuarial rate (%)
Local recurrence	4.6
True local recurrence	0.9
Second primary tumor within treated breast	1.8
Axillary relapse	1.8
Distant metastasis	1.8
Contralateral tumor	0.9

**Table 4:** Late cosmetic outcomes.

Harvard score	*n*	(%)
Excellent	107	70.3
Good	22	14.4
Fair	6	3.9
Poor	5	3.2
Not analysed	12	7.8
